# Prevalence and Risk Factors of Delayed Sputum Conversion among Patients Treated for Smear Positive PTB in Northwestern Rural Tanzania: A Retrospective Cohort Study

**DOI:** 10.1155/2017/5352906

**Published:** 2017-06-11

**Authors:** Daniel W. Gunda, Igembe Nkandala, Godfrey A. Kavishe, Semvua B. Kilonzo, Rodrick Kabangila, Bonaventura C. Mpondo

**Affiliations:** ^1^Department of Medicine, Weill Bugando School of Medicine, P.O. Box 1464, Mwanza, Tanzania; ^2^Department of Medicine, College of Health Sciences, University of Dodoma, P.O. Box 395, Dodoma, Tanzania

## Abstract

**Introduction:**

Smear positive TB carries high morbidity and mortality. The TB treatment aims at sputum conversion by two months of antituberculous. Patients who delay sputum conversion remain potentially infectious, with risk of treatment failure, drug resistance, and mortality. Little is known about the magnitude of this problem in our setting. This study was designed to determine the prevalence and risk factors of delayed sputum conversion in northwestern rural part of Tanzania.

**Methods:**

This was a retrospective cohort study involving smear positive TB patients at Sengerema DDH in 2015. Demographic data, HIV status, and sputum results at TB diagnosis and on TB treatment were collected and analyzed using STATA 11.

**Results:**

In total, 156 patients were studied. Males were 97 (62%); the median age was 39 [30–51] years. Fifty-five (35.3%) patients were HIV coinfected and 13 (8.3%) patients had delayed sputum conversion which was strongly associated with male gender (OR = 8.2, *p* = 0.046), age >50 years (OR = 6.7, *p* = 0.003), and AFB 3+ (OR = 8.1, *p* = 0.008).

**Conclusions:**

Delayed sputum conversion is prevalent in this study. These patients can potentially fail on treatment, develop drug resistance, and continue spreading TB. Strategies to reduce the rate of delayed sputum conversion could also reduce these potential unfavorable outcomes.

## 1. Introduction

Tuberculosis (TB) is the oldest infectious disease that is still a global problem causing significantly high morbidity and mortality. It is estimated that about a third of the world's population is infected with TB organism [1]. In 2015, there were more than 9.6 million people newly diagnosed with TB globally with a mortality of about 1.5 million people [2]. While it is estimated that about 80% of all TB cases occur in the 22 highest-burden countries [3], sub-Saharan Africa harbors about 70% of the words burden of TB [2].

In sub-Saharan Africa, more than 4 million people are estimated to suffer from active tuberculosis resulting in an estimated 650,000 deaths every year [4]. Tanzania is one of the 30 countries labeled as high burden countries with a total TB incidence of 164,000 people and prevalence of 275 per 100000 population [5]. A highest burden of TB is reported among densely populated regions with Dar es Salaam having the highest notification of 21.9% followed by Mwanza (9.3%), Shinyanga (6.4%), and Mbeya (6.0%) [6].

The diagnosis of TB is commonly made through sputum smear examination with smear positive patients being the most infectious ones [7] capable of infecting up to 15 people a year. Early diagnosis and prompt treatment initiation of smear positive patients are an important strategy with a goal line of smear conversion at two months of intensive phase of potent antituberculous [8]. While sputum conversion at the end of the intensive phase is regarded as an important predictor of treatment success, a significant proportion of patients on antituberculous remain positive at the end of intensive phase with some places reporting a prevalence that is in excess of 25% [9]. These patients are additionally at a high risk of developing multidrug resistance (MDR), which is a very challenging phenomenon in TB treatment [10]. The literature on the magnitude of this problem is scarce in our setting especially in the northwestern rural Tanzania. This study was therefore designed to determine the prevalence and assess the risk factors of delayed sputum conversion among adult patients treated for smear positive TB in rural setting of northwestern Tanzania.

## 2. Materials and Methods

This was a cross-sectional clinic based cohort study done at Sengerema Designated District Hospital (DDH) between August 2016 and March 2017. The study involved all smear positive TB patients who were diagnosed and initiated treatment for TB between January and December 2015 and subsequently followed up at Sengerema DDH. Sengerema DDH is one of the district level hospitals in Mwanza region situated in the western part of Lake Victoria. It serves a catchment population of about 700,000 people with a capacity of around 300 beds. Sengerema DDH runs both in- and outpatients' activities on daily basis serving between 200 and 300 patients a day. TB unit serves as part and parcel of the daily outpatient department seeing between 200 and 300 TB patients a year. Patients suspected of having TB from inpatient and outpatient departments get referred to TB unit for further diagnostic work-up. The diagnosis and treatment of TB were done according to Tanzanian TB and Leprosy guideline with sputum examination for AFB being carried on routine basis to categorize TB patients as smear positive [11]. Patients were additionally tested for HIV before commencement of antituberculous drugs. All patients received standard treatment for TB under DOTS as per reported guideline. Follow-up sputum examination was done as routine at 2 months of anti-TB and or monthly thereafter for nonsputum convertors.

A TB registry was used to identify patients and their registration number was used to trace their files. The files were then reviewed by the study team to extract the information of research interest. The information regarding age, sex, address, HIV status, date of TB diagnosis, sputum smear results and density at diagnosis, follow-up sputum smear results, and time was recorded with a special tool. The data were double-entered and cleaned using Epi data version 3.1 and STATA version 11 (Stata Corp LP, College Station, TX) was used for analysis. All continuous variables were summarized as medians with interquartile range while the categorical variables were expressed as proportions with percentages. In this study, patients who had their follow-up sputum positive after intensive phase were coded as having delayed sputum conversion as used in other studies [9, 12]. The proportion of patients with delayed sputum conversion was calculated and logistic regression model was performed to determine the odds ratio and 95% CI to find out the degree of association between the outcome of interest and its potential risk factors. In all our calculations, any risk factor had a significant statistical association if the *p* value was <0.05.

### 2.1. Ethical Consideration

The permission to conduct and publish the information from this study was sought from the Bugando Medical Centre/Catholic University of Health and Allied Sciences (BMC/CUHAS) joint ethical and research committee and Sengerema DDH administration. The files were handled by researchers alone; the patients' identifiers were not included to maintain confidentiality.

## 3. Results

### 3.1. The General Characteristics of the Study Participants

**Figure 1:**
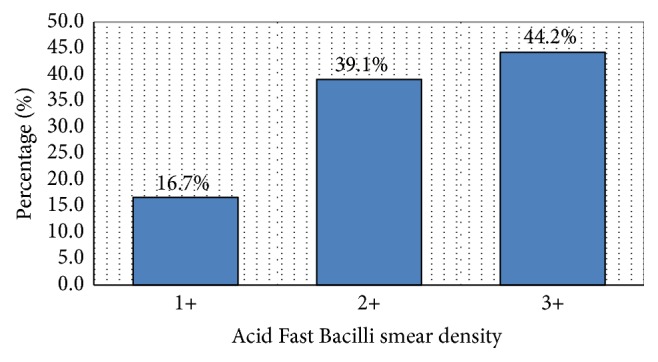
Distribution of smear positivity at treatment initiation.

In total, 156 patients were treated for smear positive TB during the study period. Most of participants were male (97) (62.0%) with a male to female ratio of 1.64 : 1 and with a median age of 39 [30–51] years. More than one-third (55) (35.26%) of the study participants were HIV coinfected. The median TB bacilli density was 2 [2-3] (Table 1).  Figure 1 summarizes the distribution of smear density at initiation of antituberculous drugs.

### 3.2. Prevalence and Risk Factors of Delayed Sputum Conversion

**Figure 2:**
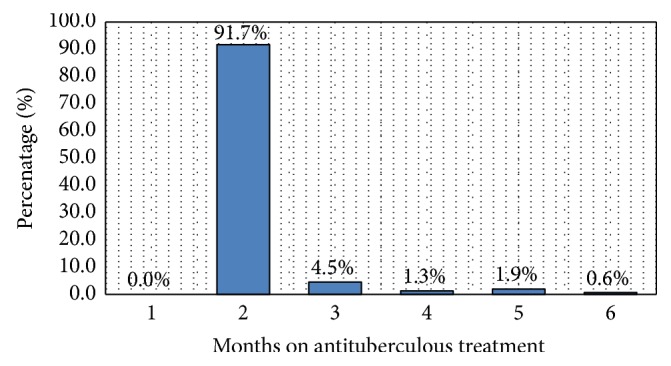
Distribution of sputum smear conversion by time on anti-TB.

In this study, a total of 143 (91.7%) patients had smear conversion within the first two months of potent antituberculous treatment, while 13 (8.3%) had delayed sputum conversion (Table 1). More details on sputum conversion by months on anti-TB are given in Figure 2. The odds of having delayed sputum conversion were strongly associated with a male gender (OR = 8.2, *p* = 0.046), age older than 50 years (OR = 6.7, *p* = 0.003), and Acid Fast Bacilli density of 3+ (OR = 8.1, *p* = 0.008). HIV coinfection was not significantly associated with delayed sputum conversion (Table 2).

## 4. Discussion

The objective of this study was to determine the prevalence and assess the risk factor of delayed sputum conversion among adult patients treated for smear positive TB in rural northwestern Tanzania. In this study, it was found that 13 (8.33%) had delayed sputum conversion beyond 2 months of intensive phase.

Our findings are comparable to findings from several other studies across the world with a varying rate of delayed sputum conversion. The prevalence of delayed sputum conversion at 2 months in the index study is similar to a prevalence of 8.1% reported from Ghana in 2015 by Acquah [13] and also similar to a prevalence 9.0% reported much earlier from India in 2006 [14] representing comparable rates of sputum conversion rates of 91.9% and 91%, respectively, at the end of intensive phase. On the other hand, slightly smaller prevalence of 7.3% of delayed sputum conversion at 2 months was reported recently from Cameroon [12]. However a much higher rate of nonsputum conversion of 25.4% was reported previously from Portugal in 2012 [9].

Several factors were investigated for their association with the odds of having delayed sputum conversion. These factors which could independently increase the risk of delayed sputum conversion include male gender, older age than 50 years, and AFB density of 3+. These findings are in agreement with those reported in Portugal in 2012. In this study, male patients were 10.8 times more likely to have delayed sputum conversion, while patients who were older than 50 years and those who had smear grade of 3+ had 4.4 and 11.7 times higher risk of delayed sputum conversions, respectively [9]. Similarly, Tiwari and colleagues indicated that the risk of smear nonconversion was increased among those with age older than 40 years (OR = 2.7, *p* = 0.003), and pretreatment bacillary load of 3+ on sputum smears (OR = 1.9, *p* = 0.037) delayed sputum conversion in addition to having a higher rate of treatment failure which tends to increase with bacilli density in sputum [15]. In their study of gender differences in distribution of TB, Balasubramanian et al. found that male patients were frequently found to have treatment failure associated with taking medications irregularly as compared to women [16] which could also explain the increased risk of delayed sputum conversion among male patients. On the other hand, preexisting comorbidities among older patients could be associated with a risk of reduced compliance to TB medications also increasing the risk of delayed sputum conversion as also suggested by other authors [17]. Other risk factors for delayed sputum conversions which were not assessed in this study include alcoholism [18], having cavities on a chest X-ray [19], and diabetes mellitus [20].

Delayed sputum smear conversion is important clinically. Apart from a possible continuity of infectiousness, prior studies have indicated that delayed smear conversion is a risk factor for both TB treatment failure and emergence of drug-resistant TB [21] and potential increase in TB mortality. In one study from Cameroon where sputum culture and sensitivity were done among those who had delayed sputum conversion, about 28.0% were found to have treatment failure [22]. Tiwari and colleagues had similar observation that there was an increased rate of treatment failure among patients with delayed sputum conversion which was linearly related to bacilli density in sputum [15]. In addition to treatment failure, Djouma et al. could show that patients with delayed sputum conversion had a significantly increased risk of mortality on anti-TB (OR = 4.4, *p* < 0.001) [12]. In another study assessing drug resistance from Taiwan, it was established that, among 34 patients who had delayed sputum conversion, 24 (70.6%) were found to have Isoniazid (INH) resistant strains of TB microbes [19].

Resistance to antituberculous drugs is an important problem with a serious threat to global control of TB [10]. The results from this study suggest that patients with delayed sputum should better be investigated for resistant strains of TB also. One important challenge is that diagnostic facilities appropriate for resistant TB are still expensive and may not be readily available for routine care [23] of patients with TB in rural Tanzania and this may also be true for most resource limited countries.

This study had a number of limitations. This was a single center study; therefore the results from the study may not be generalizable. Patients who had delayed sputum conversion could not be investigated for resistant BT due to lack of facility and the study being a retrospective study. As this was a retrospective study, missing data was common; also some parameters such as presence of cavitations and diabetes mellitus that were associated with delayed conversion in other studies could not be assessed in this study due to missing information. However, to the best of our knowledge, our study is the first study to report on the factors of delayed sputum conversion among smear positive TB patients in Tanzania.

## 5. Conclusion

The proportion of TB patients with delayed sputum conversion on potent TB treatment is still high in northwestern rural Tanzania. Patients older than 50 years and those with high AFB density have the highest risk of delayed sputum conversion. These patients can potentially fail on treatment, develop drug resistance, and continue spreading TB. Strategies to reduce the rate of delayed sputum conversion could also reduce these potential unfavorable outcomes.

## Figures and Tables

**Table 1:** Demographic and laboratory characteristics of 156 study participants.

Variables	Frequency	Median (IQR) or percentage
Sex		
Male	97	62.2
Female	59	37.8
Age	156	39 [30–51]
Age group		
≥50 years	45	28.8
<50 years	111	71.2
Resides in SGM		
Yes	18	11.5
No	138	88.5
HIV status		
Positive	55	35.3
Negative	101	64.7
Median AFB+	156	2 [2-3]
Smear conversion		
Median (months)	156	2 [2-2]
>2 months	13	8.3
≤2 months	143	91.7

AFB: Acid Fast Bacilli; HIV: human immunodeficiency virus; IQR: interquartile range.

**Table 2:** Factors associated with delayed sputum smear conversion among 156 study participants treated with potent anti-TB medications.

Variables	Delayed smear conversion		OR (95% CI)	*p* value
	No (*n* = 143)	Yes (*n* = 13)		
Sex				
Male	85 (59.4)	12 (92.3)		
Female	58 (40.6)	01 (7.7)	8.2 (1.0–64.7)	0.046
Age	38 [29–50]	53 [40–59]	1.1 [1.0–1.1]	0.013
Age group				
≥50 years	36 (25.2)	09 (69.2)		
<50 years	107 (74.8)	04 (30.8)	6.7 (1.9–23.0)	0.003
Resides in SGM				
Yes	17 (11.9)	01 (7.7)		
No	126 (88.1)	12 (92.3)	0.6 (0.1–5.0)	0.653
HIV status				
Positive	50 (34.9)	05 (38.5)		
Negative	93 (65.1)	08 (61.5)	1.2 (0.4–3.7)	0.801
Median AFB+	3 [3-3]	2 [2-3]	6.5 (1.5–27.3)	0.011
Smear bacilli density				
AFB 1+	26 (18.2)	00 (00.0)	—	—
AFB 2+	59 (41.3)	02 (15.4)	0.3 (0.1–1.2)	0.086
AFB 3+	58 (40.5)	11 (84.6)	8.1 (1.7–37.7)	0.008

AFB: Acid Fast Bacilli; HIV: human immunodeficiency virus; CI: confidence interval; SGM: Sengerema.
